# Standardized method for solubility and storage of capsaicin-based solutions for cough induction

**DOI:** 10.1186/1745-9974-10-6

**Published:** 2014-09-25

**Authors:** Michael T Costanzo, Richard A Yost, Paul W Davenport

**Affiliations:** 1 Department of Chemistry, University of Florida, Gainesville, FL 32611, USA; 2 Department of Physiological Sciences, University of Florida, Gainesville, FL 32610, USA

**Keywords:** Capsaicin, Solubility, Stability, Tussigenic challenge, UV detection

## Abstract

**Background:**

Preparation of inhaled capsaicin solutions for cough induction varies greatly from one lab to another, which creates inconsistencies between tussigenic challenge results. The addition of Tween to these capsaicin solutions provides increased solubility and stability; however, the foul taste of Tween makes inhaling the solution for any prolonged period of time unpleasant. We sought to create a standard method for preparing soluble and stable capsaicin-based solutions (in 10% ethanol/water), without the addition of Tween.

**Methods:**

Capsaicin solutions were created at concentrations ranging from 0 to 500 μM in a variety of solvent systems, with and without Tween. Samples were stored in four different environments (-20°C, 3°C, and room temperature, protected from light; and room temperature, exposed to light) to test stability. Detection of capsaicin was carried out by UV absorption. A Grubb’s test was performed on all data to remove statistical outliers.

**Results:**

Similar capsaicin concentrations were seen for solutions prepared with or without Tween (Tween provided a slight increase in solubility), with neither solvent system providing complete solubility. Of the four environments tested, storing capsaicin solutions at 3°C while protected from light afforded the greatest stability, for a minimum of 30 weeks.

**Conclusion:**

We recommend the use of a 10% ethanol/water solvent system without Tween in the preparation of capsaicin solutions for tussigenic challenges. While this solvent system does not provide complete solubility, we have detailed a method for capsaicin solution preparation that will account for this loss of solubility, while maintaining a solution that is Tween-free and safe for human inhalation.

## Background

Capsaicin (CAP) can be utilized to test the cough threshold of patients thought to have certain respiratory diseases, as well as healthy individuals [1-3]. The greatest challenges in using CAP for such tests are its low solubility in water and storage instability. Therefore, while a pure water solvent system would be ideal for such solutions requiring human consumption, water alone cannot be used since the capsaicin does not dissolve in sufficient quantities to afford meaningful testing. Hence, all of the reported studies used solutions of capsaicin in water mixed with low concentrations of organic solvents. Fortunately, CAP has been shown to be completely soluble in organic solvents, with ethanol (EtOH) providing the greatest solubility [4,5]. Further research has revealed that dissolving CAP in a mixture of EtOH and polysorbate-80 (Tween-80) allows for a higher solubility than EtOH without Tween [6-9]. Our own research suggests Tween-20 is an equivalent replacement solvent for Tween-80; thus, Tween-20 was utilized in this research.

CAP solutions containing Tween-20 have been prepared and tested on subjects with well documented dose–response relationships for cough reflex testing [10-13]. Regrettably, there was a common complaint of a bad taste from the solution, as reported in several previous studies of Tween’s use in flavor detection and awareness as the adverse stimulus [14-16]. This taste, often described as soapy and unpleasant, can be attributed to the presence of the surfactant, Tween-20 or Tween 80, in solution. Since subjects are required to inhale CAP solutions for an extended period of time, the taste of the solutions begins to cause discomfort. Although previous studies reported quantitative comparisons of CAP in solutions with Tween versus solutions without Tween [6,7], to the authors’ knowledge, there has yet to be a study that directly compares the solubility of a broad range of concentrations of CAP in 10% EtOH alone to CAP in a Tween solution. Furthermore, a comparison of CAP solubility in solutions of varying percentages of EtOH has yet to be reported. The purpose of such a comparison would be to produce CAP solutions in an EtOH-based solvent system, equivalent to those deemed fit for use in the aforementioned cough threshold tests [1,3], but without Tween.

In this study, we sought to determine (1) the percentage of EtOH yielding CAP solubility comparable to a desired concentration of CAP in Tween-20 solution, (2) the concentration of CAP dissolved in 10% EtOH required to produce analogous solubility to a desired concentration of CAP in solution containing Tween-20, (3) if the ratio of CAP present in EtOH solution to CAP present in Tween-20 solution changes with variations in CAP concentration, and (4) the shelf life of CAP in 10% EtOH. Based upon these results, we are now developing a standardized method for optimal preparation of CAP solutions for use in tussigenic challenges.

## Methods

### Chemicals and reagents

Capsaicin, pharmaceutical grade, was purchased from Formosa Labs (Taoyean, Taiwan) and stored at -20°C until use. 190 proof ethyl alcohol (95% EtOH) was purchased from Decon Labs (King of Prussia, PA). HPLC grade water was purchased from Fischer-Scientific (Fair Lawn, NJ). Tween-20 was purchased from MP Biomedicals (Solon, OH). All solutions were stored at room temperature, unless otherwise stated.

Prior to each study, a stock solution of 25 mM CAP was prepared. Initially, the CAP powder was dissolved in a solution made-up of 80:10:10 H_2_O:Tween-20:95% EtOH (hereafter referred to as "Tween solution"), but this method did not allow sufficient solubility of high concentrations of CAP. Instead, the stock solution was prepared by dissolving 76 mg (250 μmol) of solid CAP in 10 mL of 95% EtOH. Preparing such a high concentration of CAP for the stock solution allowed any error associated with variation of volume to be considered negligible. In the research presented, solutions with EtOH levels at 5, 10, 15, 20, 25, 35, 50, 65, 75, and 95% EtOH in H_2_O were prepared. Additionally, solutions of CAP were prepared at concentrations of 0, 200, 350, and 500 μM for all percentages of EtOH mentioned. Finally, a much broader range of concentrations (0, 50, 100, 150, 200, 250, 300, 350, 400, 450, and 500 μM) were prepared in 10% EtOH for more in-depth analysis. All samples were diluted to a total volume of 5 mL in triplicate.

To prepare the Tween solutions, a solution of 80:10:10 of H_2_O:Tween-20:95% EtOH (v/v/v) was initially prepared. To this Tween solution, a specified volume of 25 mM stock CAP was added to achieve the desired concentration of CAP. Solutions of CAP were prepared at concentrations of 0, 50, 150, 250, 350, and 450 μM, with a total volume of 5 mL. Similar to the CAP solutions in EtOH, the Tween solutions were prepared in triplicate.

To study the stability of CAP in the optimized solvent system, solutions at concentrations of 0, 200, 350, and 500 μM CAP in 10% EtOH/H_2_O (without Tween-20) were prepared, and left in one of four different environments: 1) room temperature and exposed to light, 2) room temperature and protected from light, 3) approximately 3°C and protected from light, and 4) approximately -20°C and protected from light. All solutions were kept in glass vials, and placed in a cardboard box to shelter them from light, except one set of vials which was directly exposed to room light for the purposes of the study. For solutions stored below room temperature (3°C and -20°C), the solutions were allowed to warm up to room temperature for two hours and then vortexed, before analysis. Visual inspections of the solutions prior to analysis indicated no turbidity. All samples were prepared in triplicate to a final volume of 5 mL.

### Determination of CAP concentration

The concentration of CAP in the solutions was measured by ultraviolet (UV) absorption spectrophotometry. A Hewlett-Packard 8450A UV/Vis spectrophotometer was utilized to monitor the absorbance at 281 nm [5]. Although the instrument offers automatic blank subtraction due to its double-beam geometry, blanks of the EtOH solvent were analyzed separately from the samples, and manual blank-subtraction was performed as the EtOH signal was negligible. However, as Tween-20 exhibited appreciable absorbance in the spectral region of interest, the double-beam capabilities of the instrument were exploited to perform blank-subtraction automatically. To ensure removal of anomalous data, a Grubb’s test with a confidence interval of *P* = 0.05 was performed on any data suspected to be a statistical outlier. All outliers were subsequently removed from the data set.

## Results

### Solubility of CAP in different solvent systems

**Figure 1:**
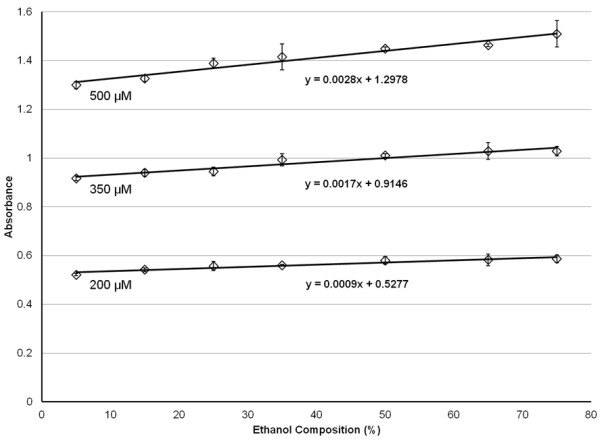
**The blank-corrected UV absorbance (λ = 281 nm) of 200, 350, and 500 μM CAP for varying compositions of ethanol.** Error bars correspond to ± 1 standard deviation.

Initially, the solubility of CAP in varying concentrations of EtOH in H_2_O was evaluated. Figure 1 displays the blank-corrected absorbance at 281 nm of CAP as a function of EtOH percentage present in the solvent. As expected, the solubility of CAP increases with increasing EtOH percentage; ramping the concentration of ethanol from 5% to 75% results in a 10 to 15% increase in solubility.

**Figure 2:**
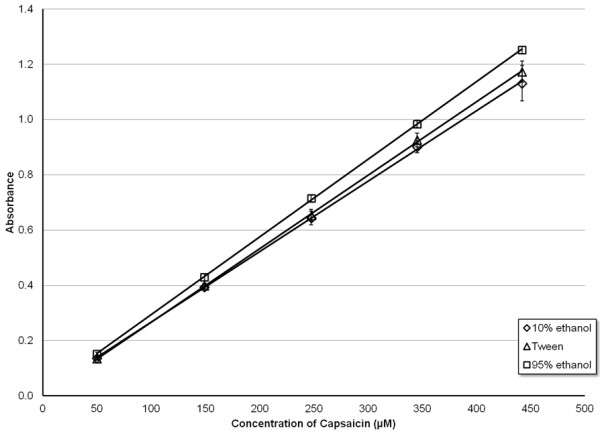
**Calibration curves for capsaicin in three different solvents: 10% EtOH, 80:10:10 H**_**2**_**O:EtOH:Tween-20 (Tween), and 95% ethanol.** Error bars correspond to ± 1 standard deviation.

The effect of a mixed solvent system containing both Tween-20 and EtOH was also investigated. Figure 2 details the CAP absorbance response curve for three different solvent systems, 10% EtOH, 95% EtOH, and 80:10:10 H_2_O:Tween-20:95% EtOH. CAP dissolved in all three solvents exhibited similar absorbance, suggesting that the solubility of CAP is similar in each solvent system tested. Despite the similarity, CAP appeared to have the greatest solubility in 95% EtOH, as evidenced by the greater slope of the response curve. As CAP is completely soluble in pure EtOH [5], the absorbance measurements obtained from CAP in the 95% EtOH solvent system were used to calculate the correct (or predicted) CAP concentrations. The average ratio of the concentration experimentally determined for CAP in 10% EtOH to the concentration of CAP in 95% EtOH (*E/P*) is around 91% (Table 1). The average ratio of the concentrations of CAP in 10% EtOH to Tween solution (*E/T*) is nearly 99%.

**Table 1:** CAP concentration in EtOH- vs. Tween-based solvent systems

**Concentrations (μM)**			
**Abs at 281 nm ± 1 std. dev. (3 replicates)**			
95% EtOH	10% EtOH	Tween	Ratio (*E/P*)
50.0 ± 2.3	45.1 ± 3.3	43.1 ± 2.7	0.90 ± 0.08
150.0 ± 0.1	135.8 ± 4.6	137.7 ± 0.8	0.91 ± 0.03
250.0 ± 0.1	226.5 ± 8.8	232.3 ± 4.4	0.91 ± 0.05
350.0 ± 0.1	317.2 ± 3.5	326.9 ± 4.4	0.91 ± 0.01
450.0 ± 0.1	407.9 ± 20.1	421 ± 8.9	0.91 ± 0.05

### Stability of CAP in 10% EtOH solutions

**Figure 3:**
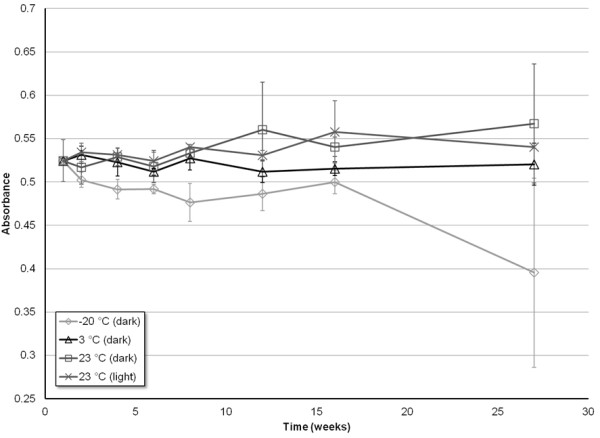
**Stability of the UV absorbance measurements (λ = 281 nm) of 200 μM CAP solutions prepared in 10% EtOH, as a function of time, for four different storage environments: -20°C (dark), 3°C (dark), 23°C (dark), 23°C (light).** Error bars correspond to ± 1 standard deviation.

**Figure 4:**
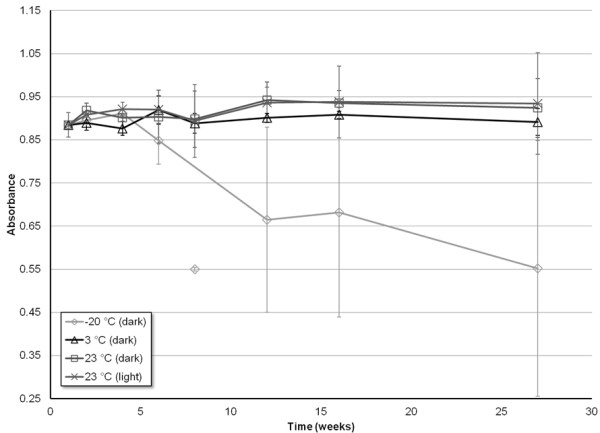
**Stability of the UV absorbance measurements (λ = 281 nm) of 350 μM CAP solutions prepared in 10% EtOH, as a function of time, for four different storage environments: -20°C (dark), 3°C (dark), 23°C (dark), 23°C (light)**. Error bars correspond to ± 1 standard deviation.

**Figure 5:**
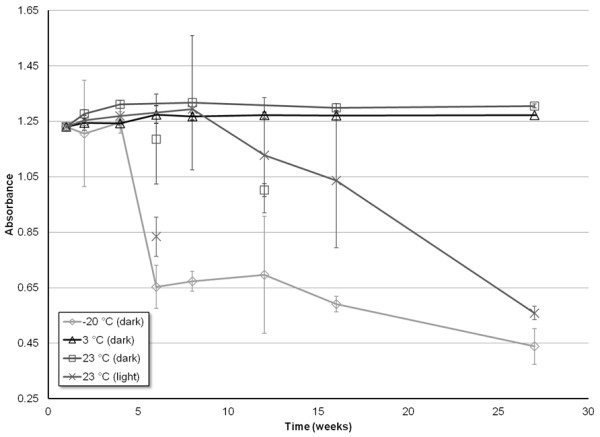
**Stability of the UV absorbance measurements (λ = 281 nm) of 500 μM CAP solutions prepared in 10% EtOH, as a function of time, for four different storage environments: -20°C (dark), 3°C (dark), 23°C (dark), 23°C (light).** Error bars correspond to ± 1 standard deviation.

To determine the stability of CAP in 10% EtOH under different environmental conditions, a time course study was conducted by monitoring the absorbance of CAP solutions. The data for each of the three concentrations monitored, 200, 350, and 500 μM CAP, are shown in Figures 3, 4, and 5, respectively.

Of all the environments, CAP stored around 3°C and sheltered from light yielded the most consistent absorbance measurements for all three concentrations tested. Furthermore, all three concentrations tested yielded absorbance measurements within error relative to the initial time point for the entirety of the seven month study.

CAP solutions stored at room temperature (~23°C), while protected from light, demonstrated a consistency in absorbance similar to when stored at 3°C. Within error, there was no decrease in absorbance, and therefore no decrease in CAP solubility from 0 to 27 weeks, for all three of the concentrations tested. The one exception to this trend was the 12 week measurement for the 500 μM concentration. This anomalous data point demonstrated a decrease in intensity that is not within error of the observed trend. Thus, both visual and statistical analysis (by utilization of the Grubb’s test) result in the data point being considered an outlier (*P* = 0.05).

CAP solutions stored at room temperature, and exposed to light, exhibit no decrease in absorbance throughout the entire seven months for concentrations of 200 and 350 μM. Conversely, for the highest concentration of 500 μM, there is a significant decrease in absorbance of CAP starting after only 2 months of storage. A single measurement for the 500 μM solution taken at the sixth week of storage (Figure 5) shows a decrease in absorbance, before returning to an absorbance similar to the week prior, and then gradually decreasing to lower absorbance as the further weeks progressed. A Grubb’s test was again utilized, and determined that this data could be considered an outlier (*P* = 0.05).The data shown for CAP stored at -20°C, while protected from light, proved to be the least consistent, in terms of both stability and error. The 200 μM solution demonstrates little to no decrease in absorbance for the first four months of storage before beginning to slowly diminish in intensity (Figure 3). The 350 and 500 μM solutions revealed a much sooner drop-off in intensity, as the absorbance began to significantly decrease after only a month (Figures 4 and 5). For the concentrations of 200 and 350 μM, the drop in absorbance intensity correlates with a dramatic increase in standard deviation of the data, rendering the data questionable at best. The measurements taken for 500 μM contain much less associated error, and can be considered more referential.

## Discussion

Studies reported by Kopec et al [6,7]. provided the groundwork for this study of CAP solutions involving the use of Tween as a solvent. However, this report involves a more comprehensive comparison of Tween and EtOH-based solutions, in terms of both solubility and stability.

Each CAP solution was comprised of only a few components (e.g. CAP, H_2_O, EtOH, and Tween-20), and each component has a different maximum absorbance in the UV spectrum. Due to a lack of sample complexity, UV/Vis detection was performed without prior HPLC separation, unlike the previous study. Therefore, time of analysis was greatly reduced. The HPLC analysis described by Kopec et al. required 12 minutes per sample [7], whereas an average sample analysis time with the method described in this work was significantly faster (~3 minutes). Furthermore, there were initial concerns over the solubility of the capsaicin, as particles large enough to scatter light were observed in solutions stored below room temperature during the time-course study; however, these particles all redissolved over the course of the 2-hour warming period and vortexing of samples before analysis. Visual inspections of the solutions prior to analysis indicated no turbidity. The use of a spectroscopic technique permits non-destructive and rapid analysis of the samples, but affords less selectivity than would be achieved with a supplementary HPLC separation step [17]. Future studies utilizing HPLC separation or other analytical techniques could provide confirmation of these results.

From the results of our study, it can be concluded that CAP solutions that are similar in solubility and stability to those with Tween-20 can be prepared using purely EtOH-based solvents, yet lack the foul taste associated with Tween. Kopec et al [6,7]. determined that utilization of a solvent system of Tween-80, ethanol, and water for preparation of CAP solutions yields better solubility and stability than a solvent system comprised of ethanol and water without Tween. However, the concentration of ethanol in their reported solutions was only around 1%. The primary purpose of our study was determine if preparing capsaicin solutions in similar fashion to Kopec benefitted from Tween addition enough that its use would be recommended. As the reported preparation of the Tween solutions did not involve any separation step, we wanted to analyze the solutions as they would be prepared clinically. In this respect, any aspect of dissolution that may result in suspension/dispersion of CAP would accurately reflect clinical implications when conducting the test. As discussed in more detail below, no turbidity was observed in samples ready for analysis following their warming to room temperature and vortexing. The present work suggests that Tween-20 yields a less than 2% increase in solubility over a solvent system of ethanol and water, where the ethanol concentration is 10% rather than 1% by volume. Furthermore, regardless of the inclusion or exclusion of Tween-20, solvent systems safe for human consumption (i.e., less than 20% ethanol by volume) generally demonstrate CAP concentrations below the predicted concentration; therefore, neither allows complete solubility.

As noted in Figure 1, there is a modest increase in solubility as the composition of EtOH is increased from 5 to 75%; however, this difference can be easily corrected for by dissolving additional CAP into the solution, as described in more detail later. Thus, lower percentages of EtOH are recommended to allow safe human inhalation, approximately 20% or less EtOH. Therefore, we have chosen to use 10% EtOH solutions to decrease the amount of EtOH solvent in the inhaled vapor. Furthermore, the concentration of CAP used for the tussigenic challenge does not typically exceed 500 μM; [12] therefore, an increase in variation between high and low EtOH compositions at higher concentrations of CAP does not become a factor.

For solutions in 10% EtOH, there is a 10% decrease in solubility relative to the predicted solubility (Table 1); predicted concentration is determined from absorbance measured for CAP dissolved in the 95% EtOH solvent system. This can be overcome by increasing, by 10%, the amount of CAP initially dissolved in solution. Accordingly, a suggested standardized sample preparation for CAP solutions in an EtOH-based solvent system is detailed below.

### Recommendations of preparation of CAP solutions in EtOH solvent system

In this study, we used 95% EtOH and accounted for the impurity in our dilutions; however, the following guideline uses 100% EtOH to allow for easier calculation. *Note: The ethanol used must be safe for human consumption and the capsaicin pharmaceutical grade.*

**Step 1.** Dissolve solid CAP in 100% anhydrous EtOH to create a concentrated CAP stock solution. Dissolving 0.7635 g of solid CAP (2.5 mmol) in 100 mL of 100% EtOH will produce a 25 mM stock solution of CAP.

**Step 2.** Prepare the solvent for the inhaled CAP solutions by diluting 100% EtOH with H_2_O. To create 10% EtOH solutions, combine 100 mL of 100% EtOH with 900 mL of H_2_O.

**Step 3.** Add the CAP stock to the 10% EtOH to produce the desired concentration of CAP in 10% EtOH, using the following equation:

(1) $$V_{\text{stock}}\;=\;\left(\frac{C_{\text{cap}}\cdot V_{\text{cap}}}{C_{\text{stock}}}\right)1.1$$

Where *C*_*stock*_ and *V*_*stock*_ are the concentration and volume of the CAP stock solution, and *C*_*cap*_ and *V*_*cap*_ are the concentration and volume, respectively, of the desired CAP solution in 10% EtOH. Recall that the initial amount of CAP added needs to be increased by 10% to account for the decrease in solubility associated with a 10% EtOH solvent. Equation 1 may be utilized to prepare any desired CAP solution, or Table 2 may provide a quick reference for specific concentrations.

**Table 2:** Recommended preparation of 1 L CAP solutions at a range of useful (tussigenic) concentrations

**Desired concentration of capsaicin in solution (μM)**	***Volume of 25 mM capsaicin stock (mL)***	***Volume of 10% ethanol (mL)***
5	0.22	999.78
10	0.44	999.56
20	0.88	999.12
40	1.76	998.24
100	4.40	995.60
150	6.60	993.40
250	11.00	989.00
375	16.50	983.50
500	22.00	978.00

### Stability of CAP in 10% EtOH solutions

The stability of solutions of CAP prepared with Tween-80 have been previously reported [7], hence, we did not repeat a similar investigation. Instead, we focused solely on the stability of CAP solutions prepared in 10% EtOH, which were diluted in HPLC-grade water rather than saline solution.

The most pertinent findings from this study show that the greatest stability may be achieved by storing the specified solutions of CAP in a light-free environment, and at a temperature of approximately 3°C. Under such conditions, the solutions of CAP in 10% EtOH remained stable, over a range of concentrations, for a minimum of 30 weeks (nearly 8 months, the duration of the storage study), as seen in Figures 3 – 5. Additionally, the highly concentrated stock solutions of CAP prepared in pure EtOH remained stable for at least a year (the maximum time tested) under the same conditions. This increase in stability of non-Tween CAP solution compared to that shown in the previous study [6,18] may be attributed to either the use of distilled water as the dilution solvent rather than saline solution, or the increase in EtOH composition. Use of saline may destabilize the CAP solutions causing them to degrade quicker. Alternatively, the presence of a higher percentage of EtOH in solution may provide greater stabilization of CAP. The drastic drop in solubility of solutions stored at -20°C might be due to the low temperature causing some CAP to precipitate out of solution and adsorb to the walls of the container, and then not redissolve once the solution is brought back up to room temperature and vortexed.

## Conclusions

In this study, we demonstrated that CAP solutions prepared in a 10% EtOH solvent system are nearly as soluble as CAP solutions prepared in a Tween-incorporated solvent system. Although neither solvent system allows complete solubility of CAP, the concentrations that can be achieved are quite adequate for tussigenic challenges. Therefore, to avoid the foul taste of Tween and still efficiently prepare soluble CAP solutions in 10% EtOH, a simple equation may be followed (Equation 1). In this work, we have provided the groundwork for creating a standardized approach to preparing CAP solutions for use in tussigenic challenges. Until further refined methods are established, the preceding preparation steps should be taken to achieve maximum solubility of CAP in solution. Similarly, for optimal storage, keep solutions shielded from light and at a temperature of around 3°C.

Ongoing studies were performed utilizing the Tween-free methods for preparing capsaicin solutions to elicit cough (unpublished results). Tween-free 50–500 μM capsaicin solutions were prepared in 90% physiological saline and 10% ethanol. These solutions were used to elicit cough in normal subjects. These solutions were used immediately after preparation and after storage for 1–4 months, either refrigerated or at room temperature. No differences as a function of vehicle solution (90% physiological saline, 10% ethanol vs 80% physiological saline, 10% Tween 20, and 10% ethanol), storage time, or storage temperature were observed for capsaicin elicited cough threshold, cough number, Urge-to-Cough Threshold, and Urge-to-Cough sensitivity compared to prior studies [13,19,20] for equal concentrations of capsaicin. There appears to be fewer vehicle elicited coughs with 90% physiological saline and 10% ethanol solutions compared to 80% physiological saline, 10% Tween 20, and 10% ethanol solutions. Future studies are required to systematically compare the cough response to capsaicin using these vehicle (90% physiological saline, 10% ethanol vs 80% physiological saline, 10% Tween 20, and 10% ethanol) solutions.

## Competing interests

The authors declare that they have no competing interests.

## Authors’ contribution

MC participated in the conception, design, analysis, interpretation, drafting the manuscript, critically reading it and final approval of the submitted manuscript. RY participated in the design, analysis, interpretation, critically reading the manuscript and final approval of the submitted manuscript. PD participated in the conception, design, analysis, interpretation, critically reading the manuscript and final approval of the submitted manuscript. All authors read and approved the final manuscript.
